# Photonic Methods for the Assessment of Lesion Activity

**DOI:** 10.3390/diagnostics16121908

**Published:** 2026-06-19

**Authors:** Daniel Fried

**Affiliations:** Department of Preventive and Restorative Dental Sciences, University of California, San Francisco, 707 Parnassus Ave, San Francisco, CA 94143, USA; daniel.fried@ucsf.edu

**Keywords:** lesion activity, dental caries, optical coherence tomography, fluorescence imaging, SWIR imaging, thermal imaging

## Abstract

**Background/Objectives:** This review describes the advantages of new photonic-based approaches for assessing the activity of caries lesions. Many lesions have been arrested or are non-carious developmental defects, such as fluorosis, which do not require intervention. New methods are needed to assess lesion activity and avoid unnecessary removal of the tooth structure. **Methods:** At present, there are no reliable methods for assessing lesion activity in vivo. Nondestructive optical monitoring of lesion structure and the changes in light scattering that occur during drying offer the potential for lesion activity assessment during a single examination. Since optical diagnostic instruments exploit changes in the porosity and the permeability of the lesion, they have the potential to assess whether lesions are active and expanding or arrested and undergoing remineralization. Optical coherence tomography (OCT), Raman imaging and fluorescence loss, thermal and short-wavelength infrared (SWIR) reflectance measurements during lesion dehydration with forced air are presented. **Results:** Clinical studies have shown that optical coherence tomography is capable of showing distinct structural differences between active and arrested lesions on coronal and root surfaces. Differences in the kinetics of dehydration measured using reflectance measurements at SWIR wavelengths coincident with water absorption bands also show great potential. **Conclusions:** OCT and dehydration imaging at SWIR wavelengths have great potential for assessing lesion activity since they can also be used for caries screening, are safe for frequent monitoring and do not require the application of external agents.

## 1. Significance of Lesion Activity Assessment

During the past century, the nature of dental decay or dental caries in the US has changed markedly due to the introduction of fluoride to drinking water, the advent of fluoride dentifrices and rinses, and improved public awareness. In spite of these advances, dental decay continues to be the leading cause of tooth loss in the US [1,2]. Older adults are at risk of root caries which has increasing incidence with our aging population [1,2]. Caries lesions are routinely detected in the US using visual/tactile (explorer) methods coupled with radiography. Clinical and radiographic methods lack sensitivity for early lesions and by the time the lesions are radiolucent they have often progressed well into the dentin, at which point surgical intervention may be necessary [2]. The caries process is potentially preventable and curable. If carious lesions are detected early enough, it is likely that they can be arrested by non-surgical means through chemical intervention and dietary changes [2]. Accurate determination of the degree of lesion activity and severity is critical for accurate diagnosis and effective clinical management and decision-making [3]. Most detected lesions have been arrested or are non-carious developmental defects, such as fluorosis, which do not require intervention [2]. New methods are needed to assess lesion activity and avoid unnecessary removal of the tooth structure. Measurements of lesion structure and the optical and physical changes to lesions that occur during drying offer the potential for lesion activity assessment during a single examination. Since optical diagnostic instruments exploit changes in the porosity and the permeability of the lesion, they can provide a more accurate diagnosis of the current “state of the lesion”, i.e., whether the caries lesion is active and expanding or arrested and undergoing remineralization.

Lesions are not typically visible on radiographs until decalcification has exceeded 30% [4,5], while major changes in the light scattering of caries lesions occur with less than 5% decalcification [6,7]. Clinical studies have demonstrated that SWIR imaging and OCT are capable of higher diagnostic performance than radiography for the detection of lesions on the proximal and occlusal surfaces [8,9]. Such methods can be used as frequently as needed since there is no risk of exposure to ionizing radiation. The risk of exposure to low levels of ionizing radiation is poorly understood and even greatly reduced levels of radiation exposure may still pose a significant risk, especially for children and pregnant women [2].

Caries management in dentistry has evolved from extraction to surgical restoration and is now slowly progressing to more conservative management, including remineralization [2]. Clinicians have been slow to embrace non-surgical methods, often because reliable markers of remineralization are lacking. Early and reliable diagnostic criteria of caries activity are necessary for informed clinical decisions. With early identification and the ability to monitor changes in remineralization, minimally invasive interventions are more likely to be implemented by clinicians. At present, there are no reliable methods for assessing lesion activity in vivo. International Caries Detection and Assessment System (ICDAS) criteria [10] have been introduced to assess lesion activity, but this approach ultimately relies on visual and tactile examination that is highly subjective and is not reliable, especially for hidden or suspicious occlusal lesions [11,12]. The penetration of fluorescent dyes [13] and bioluminescent chemical agents [14,15] has been proposed for assessing lesion activity; however, such approaches require the use of external agents and their effectiveness has yet to be established on all tooth surfaces or independently investigated.

The first photonic approach for assessing lesion activity was the idea of taking QLF measurements before and after lesion dehydration [16]. SWIR reflectance measurements and thermal measurements during dehydration soon followed. OCT images show structural differences between active and arrested lesions and match the images shown in PLM, microCT and TMR [7]. Raman measurements show differences in the crystallinity and mineral quality in dental hard tissues and therefore have the potential to assess lesion activity, though there have not been any specific studies on lesion activity assessment [17].

## 2. Literature Search

Even though the published work in this area is limited, searches on the PubMed database were carried out for each method, combining the keywords dental, dentistry, caries, and lesion activity for all dates. References specifically related to lesion activity assessment and the potential for lesion activity assessment were included in this review. Only earlier references that were already described in prior review articles were excluded.

## 3. Conventional Methods of Lesion Activity Assessment

Conventional clinical diagnosis of caries is based on a combination of visual and tactile exams that are highly subjective [18,19]. Color changes are due to the accumulation of stains in remineralized/arrested caries lesions that have no direct link to the extent of lesion severity, and arrested lesions and developmental defects often appear unstained [20]. The ICDAS II caries classification system [18] incorporates clinical visual–tactile examination criteria introduced by Nyvad [19]. The optical appearance, including color, surface reflectivity, and texture, is used, where active lesions appear chalky and rough while arrested lesions appear smooth and shiny. Investigators have not developed a reliable relationship between appearance and lesion activity [18,19]. Even though most experts agree that active lesions are soft, tactile hardness assessments remain subjective and lack reliability. There is also the potential for permanent damage to any intact protective lesion surface layer from sharp instruments and the use of a dull explorer is recommended if any is used, particularly with lesions on root surfaces. Histological analyses for lesion assessment such as transverse microradiography (TMR) and polarized light microscopy (PLM) require destruction of the tooth and are not suitable for use in vivo.

Longitudinal monitoring with radiographical methods is not ideal since it is not sensitive to small changes in lesion structure and mineral density and cannot be used for frequent monitoring. Moreover, caries progression does not proceed at a constant and predictable rate and can change rapidly with changes in diet and hygiene. Nondestructive visualization of the lesion structure using microCT appears to be the most promising gold standard for assessing lesion activity nondestructively [21,22,23]. MicroCT cannot be used in vivo; however, optical coherence tomography (OCT) can also be used to provide high-resolution images of the lesion structure and it can be used clinically [7].

## 4. Fluorescence and Quantitative Light Fluorescence (QLF)

Fluorescence imaging methods for caries detection were developed nearly 40 years ago [24,25]. Several fluorescence-based imaging systems have been introduced commercially that utilize green and red fluorescence. The green is due to the excitation of fluorophores in the underlying collagen of dentin by illumination with blue light and light scattering as areas of demineralization attenuate the emitted yellow/green fluorescence exiting the surface [24]. Therefore, lesion contrast is due to the loss of fluorescence from lesion areas. Excitation wavelengths have varied from 370 nm to 488 nm, and blue laser diodes at 405 nm are typically employed. Fluorescence images provide increased contrast between sound and demineralized tooth structures and avoid the interference caused by specular reflection or high glare from the tooth surface, which can interfere with visual detection of white spot lesions. A major limitation is that stains and plaque also fluoresce strongly, greatly confounding detection [25,26]. The degree of enamel demineralization in lesion areas has been correlated with the loss of the yellow/green fluorescence intensity [27]. This approach is called the QLF (quantitative light fluorescence) method. An empirical relationship between overall mineral loss (ΔZ) and fluorescence loss (ΔF) was established and can be used to monitor lesion progression on enamel surfaces [28]. The gold standard for quantifying lesion severity and tooth surface and subsurface demineralization is microradiography. Lesion severity is typically reported as the product of the volume % mineral loss and the lesion depth, ΔZ (vol.% × µm). Therefore, it is advantageous to be able to report a similar measure using optical methods. It is important to point out that QLF researchers report changes in fluorescence radiance (ΔF%) calculated as follows, ΔF _Ref_ = (F_Ref_ (demin)/F_Ref_ (sound)) × 100, for comparison with the ΔZ value measured with TMR [29]. Excellent correlation has been established between ΔF and ΔZ for shallow uniform artificial lesions on smooth surfaces [30].

QLF has also been used to quantify and measure remineralization both in vitro and in vivo [31]. Studies have shown optical changes in the fluorescence intensity of white spot lesions around orthodontic brackets in vivo after exposure to a remineralization solution or removal of a plaque retention device [32]. However, since QLF cannot produce an image of the internal structure of a lesion, it cannot be used to determine if actual mineral repair has taken place or if the lesion simply eroded away after removal of the bracket from abrasive action.

In vitro QLF studies of lesion dehydration with forced air have shown that the change in ΔF can be used to differentiate between active and arrested white spot lesions on enamel surfaces [16]. Ando et al. [33] carried out the only clinical study using this approach to measure lesion activity. They examined, *n* = 24 white spot lesions on the smooth surfaces of 23 children. Lesions were air dried for 15 s and the change in the fluorescence loss from the lesion area was calculated for 0, 5, and 15 s time points. The fluorescence loss was lower for the inactive vs. the active lesion areas; however, the difference was not significant. A major limitation of the study was that clinical examination using ICDAS [18] and Nyvad’s criteria [19] was used to assess the activity of the lesions for comparison, rather than a less objective method such as optical coherence tomography or the analysis of teeth extracted after examination using PLM, TMR or microCT. QLF studies have focused on smooth-surface enamel lesions, and the potential for assessment of occlusal or root caries lesions using QLF is problematic. The fluorescence signal depends on the total enamel thickness and imaging angle, and highly convoluted surfaces, such as the occlusal surfaces of a tooth, hinder the diagnostic capabilities of QLF [30,34].

An even more popular fluorescence approach relies on the red fluorescence from porphyrins to detect caries lesions, particularly those hidden beneath the occlusal surface. Bacteria produce significant amounts of porphyrins and dental plaque fluoresces upon excitation with red light [35]. This approach has led to the development of several fluorescence-based caries detection devices, including the DIAGNOdent [36]. Cariogenic bacteria do not produce porphyrins, and the fluorescence is due to other noncariogenic bacteria and blood breakdown products that are present in the stains that may accumulate below the surface in porous lesion areas; therefore, this approach is not suitable for use to quantify lesion activity.

High-resolution dye-enhanced fluorescence imaging has also been used to differentiate active from arrested lesions [13]; however, this approach requires cross-sectioning of the teeth and is only useful for ex vivo studies. Fluorescent dyes can also be used to measure the local pH and potentially monitor the activity of caries lesions since active lesions and the presence of a cariogenic biofilm generate acid that lowers the local pH [37,38].

Another approach is the introduction of calcium-sensitive proteins that generate bioluminescence [39,40]. This approach is both a chemical and optical approach, requiring the application of the agent followed by the imaging of the luminescence, with more active lesions releasing more calcium and producing more luminescence. Active and arrested caries lesions need to be compared using this approach, while only sound and lesion areas have been compared to date. Further research is needed to determine if this approach can be used effectively in vivo.

## 5. Optical Coherence Tomography (OCT)

**Figure 1:**
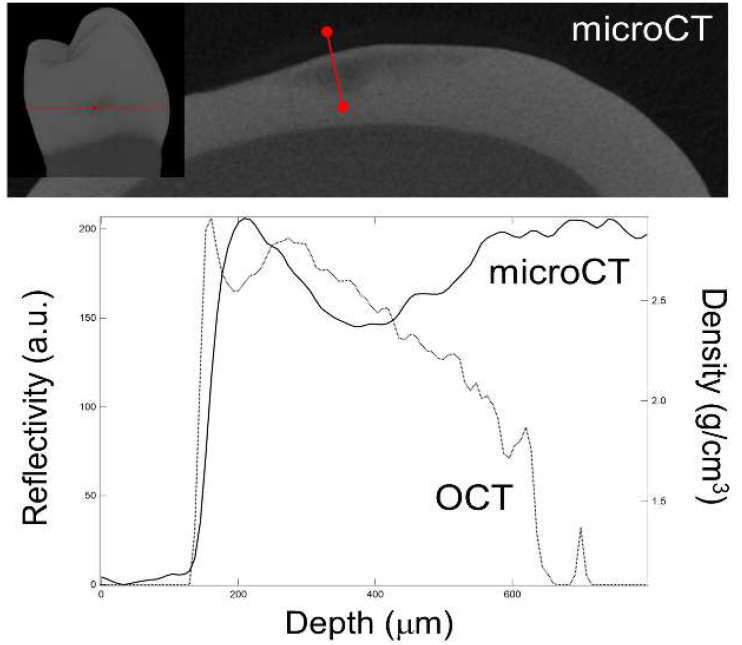
(**Top**) Cross-section extracted from a microCT image of an arrested interproximal lesion at the position of the red dotted line. (**Bottom**) Line profiles of reflectivity and density extracted from matched OCT and microCT images. From reference [21].

**Figure 2:**
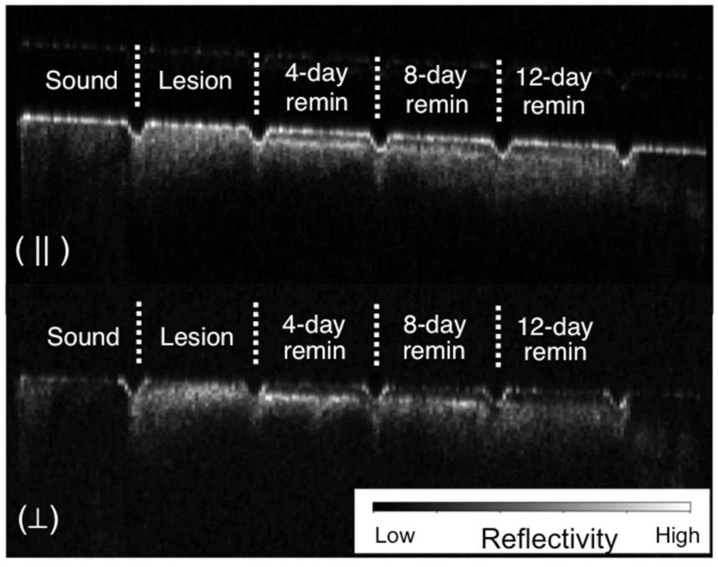
PS-OCT b-scan images of a bovine enamel block showing the sound (protected) regions located on the extreme left and right sides of the sample and lesion areas exposed for increasing periods of time to the remineralization solution: 0 (original lesion), 4, 8, and 12 days. The (||) image represents the light reflected in the co-polarization while the (⊥) image is the cross-polarization image which was used for analysis in these studies. The incisions are ~ 100 µm deep and separated by 1.4 mm. From reference [45].

**Figure 3:**
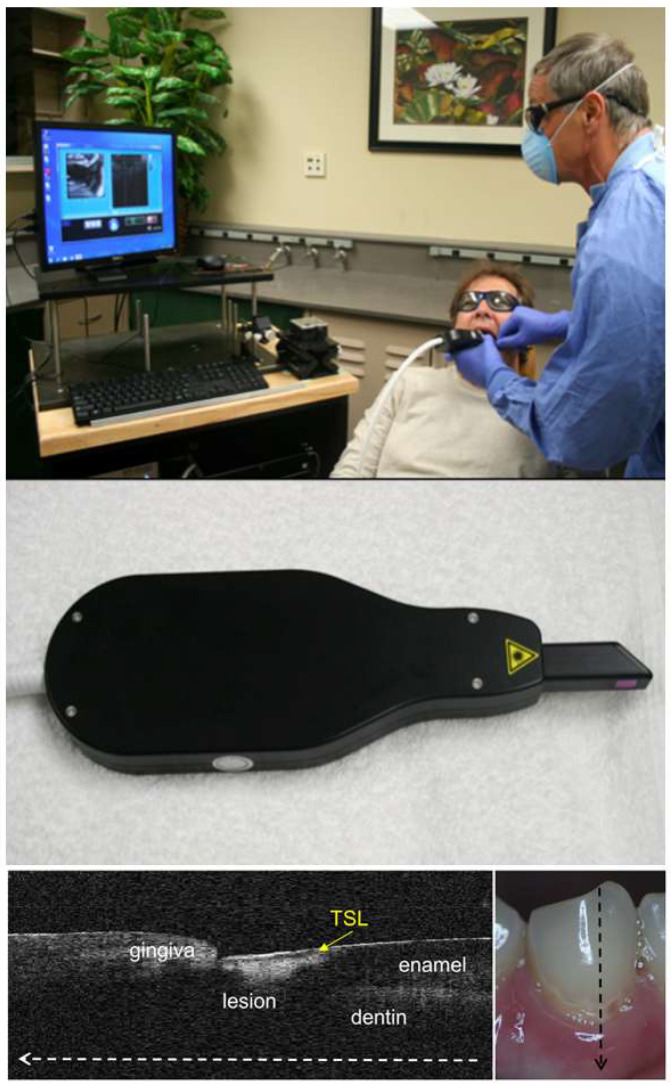
Clinical OCT system and handheld 2D scanner, along with a CP-OCT image of an arrested lesion on the tooth buccal surface acquired in vivo. The CP-OCT b-scan matches the position of the dashed arrow. The dark band of reduced reflectivity near the surface of the lesion is the TSL. From reference [53].

Optical coherence tomography (OCT) is ideally suited for dental imaging due to the high transparency of dental enamel at SWIR wavelengths between 1000 and 1400 nm [7,41]. OCT uses low-coherence light to generate cross-sectional images of the internal structure of caries lesions on both coronal and root surfaces. OCT can be used to measure the lesion structure with high resolution and determine the depth of most lesions in the enamel above the dentin–enamel junction (DEJ) [7]. OCT is typically employed at 1300 nm, where sound enamel has its greatest transparency. Demineralized enamel has a scattering coefficient 1–2 orders of magnitude higher than sound enamel; therefore, lesions are visible with high contrast at 1300 nm [6]. Upon remineralization, mineral fills the pores in the demineralized enamel. Lesion remineralization begins at the surface of active lesions forming a highly mineralized surface zone, and the enamel in that zone can regain its transparency when the mineral volume approaches that of sound enamel. The highly mineralized surface zone can be monitored with TMR, PLM, microCT and OCT [21,42,43]. Arrested lesions have a well-defined highly mineralized surface zone that is formed by preferential deposition of new mineral near the lesion surface. The highly mineralized surface layer appears more transparent than the lesion body, as shown in Figure 1, Figure 2 and Figure 3, and can be referred to as a transparent surface layer (TSL). The TSL inhibits the diffusion of fluids into and out of the lesion. Active lesions lack a highly mineralized surface layer. MicroCT cannot be used clinically; however, OCT can be used to measure the thickness of the TSL and monitor its formation in vivo [21,44]. Therefore, the presence of a TSL at the surface of lesions in microCT and OCT images is a key indicator of lesion arrest and can potentially serve as a surrogate gold standard for lesion activity. Several studies have shown that the TSL measured with OCT and the highly mineralized surface zone measured with TMR and microCT correlate well with lesion activity or permeability [7]. Studies also indicate that the dehydration rate decreases significantly with increasing TSL thickness, which explains why the dehydration imaging approaches presented in the other sections can also be used [7,21]. Almost all caries lesions, active or arrested, have outer zones of higher mineral content observable in TMR or microCT images. However, in order for a surface zone to be “transparent” in OCT images, the mineral content has to be sufficiently high, close to that of sound tissues [21]. Figure 1 shows a microCT cross-section of an arrested proximal lesion along with matching OCT and microCT profiles. The surface zone has a mineral density equal to sound enamel, indicating that it is arrested.

Polarization-sensitive (PS-) and cross-polarization (CP-) OCT systems offer improved performance over conventional OCT systems since the strong surface reflections at tooth surfaces are reduced and demineralization appears with higher contrast. These advantages are particularly important for improving the contrast of demineralization and the visibility of the TSL [21,46]. Several studies have shown that only measurement of the reflectivity in the cross-polarization state is necessary, requiring a less-expensive CP-OCT system that employs a single detector rather than the two detectors needed for a PS-OCT system [7]. PS-OCT images are typically used to measure differences in the birefringence of tissues, and dental hard tissues are birefringent. However, several studies have shown that higher contrast of demineralization can be obtained due to polarization scrambling caused by the highly scattering areas of demineralization that produce large increases in the reflectivity in the cross-polarization image [7]. The large difference in scattering between sound and demineralized areas results in a strong rise in reflectivity in both co- and cross-polarization images; however, the contrast between sound and demineralized areas is significantly higher in cross-polarization images. The reflectivity of tooth surfaces is extremely high due to the high refractive index of dental enamel, which is 1.63, and surface reflection is greatly reduced in the cross-polarization image. In the co-polarization image, the surface reflection typically exceeds the reflectivity from the surface of the lesion by several orders of magnitude, and it can mask the presence of the important TSL at the surface. The surface reflection can also be reduced by using index-matching agents or by varying the angle of incidence, but this is less practical to exploit due to the highly convoluted occlusal surfaces of teeth [7]. Another major advantage of removing the strong surface reflection is that the reflectivity can be directly integrated over the lesion depth to give a quantitative measure of lesion severity, regardless of the tooth’s topography. Such a measure can be used to better monitor changes in lesion severity over time. The integrated reflectivity over the lesion depth, ΔR, can be compared to the integrated mineral loss over the lesion depth, ΔZ, obtained from TMR or microCT [28]. Most studies using OCT assess the severity of demineralization by simply measuring the lesion depth or the increase in reflectivity [47,48,49,50,51] rather than integrating the reflectivity over the depth. More importantly, such measurements can be used to determine the presence of a TSL by automated algorithms to assess lesion activity [7].

Developmental defects, such as hypomineralization typically caused by fluorosis, have large TSLs and resemble arrested lesions; such defects do not require intervention [3,52]. Developmental defects typical of mild fluorosis have thick TSLs that have a higher mineral content than the hypomineralized body of the defect and can be resolved in OCT images [7,21].

Differences in the co-and cross-polarization images of demineralization can be seen using in vitro simulated caries models of demineralization and remineralization [7]. Figure 2 shows PS-OCT images of four areas of demineralization on a bovine enamel block with multiple treatment windows that had been exposed to a remineralization solution for 0, 4, 8 and 12 days [45]. The first area (0 days) represents an active lesion before exposure to the remineralization solution, and a thick layer of increased reflectivity is visible in both co- and cross-polarization images, representing the body of the lesion. After 4 days of exposure to the remineralization solution, the reflectivity decreased in the upper half of the lesion body as new mineral filled the pores in the lesion, forming a TSL. The mean integrated reflectivity measured with OCT and the integrated mineral loss measured with TMR for the ten bovine blocks both showed progressive loss from 0 to 12 days due to remineralization in the lesion areas [45]. This appears in Figure 2 as a gradual decrease in the reflectivity from the lesion body over time. Note the strong surface reflections visible in the co-polarization image shown in Figure 2. The strong surface reflection is orders of magnitude larger than the reflectivity from the body of the lesion, while in the cross-polarization image the surface reflection is barely visible and does not contribute to the integrated reflectivity over the lesion depth. The surface reflectance is only visible in the cross-polarization image of Figure 2 due to crosstalk in the polarization-maintaining fiber; otherwise, it would be eliminated. All the measurements needed to quantify the lesion severity, the lesion depth, the thickness of the TSL and the integrated reflectivity over the lesion depth (ΔR) can be determined solely from cross-polarization images. The co-polarization image is only useful to better show the exact position of the tooth surface, which is more clearly visible in that image. TSLs can be clearly resolved in clinical OCT images. A clinical OCT image is shown in Figure 3, along with the CP-OCT system and the 2D scanning handpiece that was used in that study [53].

More recent PS-OCT studies have investigated the potential of degree of polarization imaging for the detection of dental caries [54,55,56,57] and for monitoring the degree of demineralization and remineralization [56]. Polarization axis orientation has also been investigated [58]. It is unclear whether these more complex PS-OCT approaches perform better than CP-OCT for enhancing the contrast of demineralization or for better resolution of the structure of caries lesions, and they require even more expensive PS-OCT systems [59]. The severity of demineralization has also been quantified using calculation of attenuation coefficients [59,60]; however, that is problematic due to the complexity of the lesion structure and the varying mineralization of the different zones in lesions.

**Figure 4:**
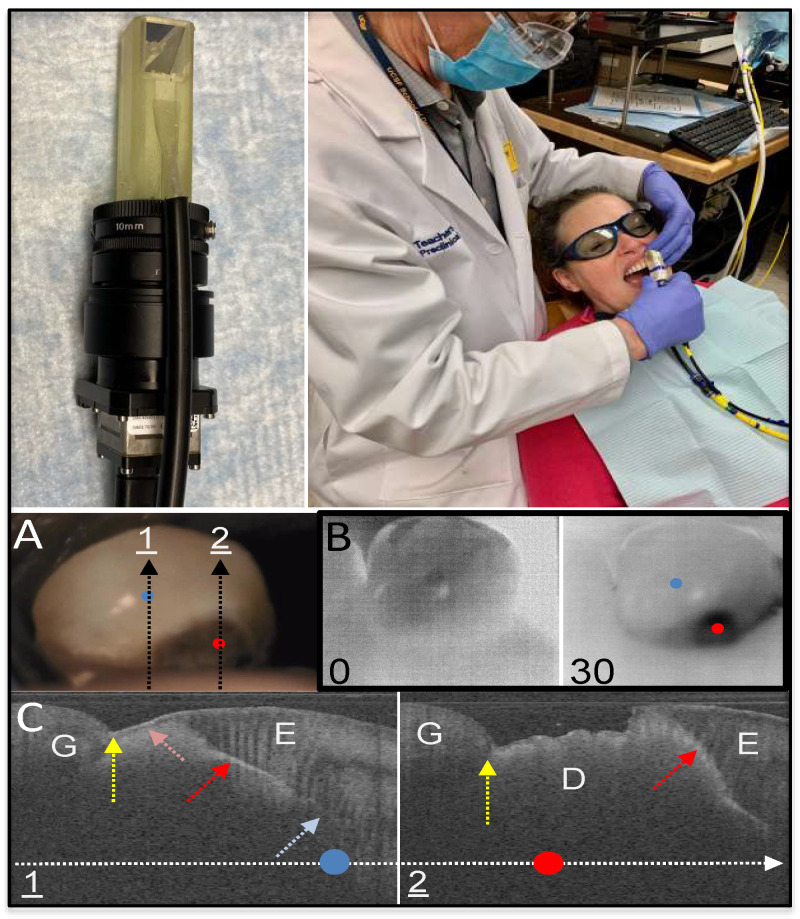
Clinical thermal imaging handpiece, along with in vivo color, thermal and CP-OCT images of a root lesion. (**A**) Color image of the tooth: the two arrows show the path of the CP-OCT images, and the colored markers show the respective positions in the CP-OCT scans shown in (**C**), where the red circle is in the lesion area and the blue circle is in sound enamel (E). Two thermal images extracted at 0 and 30 s time intervals during drying are shown in (**B**). Two CP-OCT scans labeled 1 and 2 are shown in (**C**), taken at the position of the arrows in (**A**). The yellow arrow points to the position between the exposed root and the gingiva (G). The light blue arrow points to the DEJ. In the first CP-OCT image the enamel, (E) is intact and none of the root surface is exposed; the large and deep lesion extends under the enamel (red dotted arrow). A surface zone is present on the exposed lesion surface (pink dotted arrow). The second CP-OCT scan (2) shows that the enamel has been lost and there is a large area of exposed demineralized dentin (D) and that the lesion has spread under the adjacent enamel. From reference [61].

Even though light scattering is much higher in sound dentin due to the higher scattering of dentin, OCT can be used to discriminate between sound and carious dentin and cementum [7]. Cementum has a lower reflectivity than dentin in OCT images, making it possible to differentiate dentin from cementum and monitor the loss of cementum on root surfaces [7,61]. Several studies have demonstrated that CP-OCT can be used to quantify the severity of demineralization on root surfaces with high resolution [7]. Root caries are shallow and 1–2 mm of optical penetration can be achieved at 1300 nm, which is sufficient for the diagnosis of most lesions [7]. OCT can also be used to measure the remineralization of dentin [7] and measure the thickness of TSLs on arrested lesions to assess the activity of root caries lesions clinically [61]. Dentin and cementum contain a high percentage of collagen and demineralized dentin shrinks or contracts with loss of water. Such shrinkage can be easily measured using OCT [61,62]. The formation of a highly mineralized surface zone and TSL upon remineralization and arrest of active root caries lesions also prevents shrinkage during dehydration. Measurement of shrinkage before and after drying is yet another way OCT can be used to assess the activity of root caries lesions. Therefore, structural features such as the presence of a highly mineralized transparent surface zone and the occurrence of shrinkage, both of which can be measured with OCT, can potentially be used in vivo to assess lesion activity. CP-OCT images of active and arrested root caries images are shown in Figure 4C, acquired during a clinical study assessing the use of both CP-OCT and thermal imaging to assess lesion activity [61].

## 6. Thermal Imaging

Caries lesions are porous and retain water when well-hydrated in the oral environment, and that water is lost to evaporation when dried with forced air, which is a highly endothermic process. The heat of the vaporization of water is large and the loss of water due to evaporation lowers the temperature at the lesion surface. Significant changes in surface porosity occur when lesions are arrested; therefore, a drop in surface temperature or the thermal emission from the lesion surface during drying can be monitored with a thermal camera to differentiate between sound and carious tissues and active and arrested lesions. The less-permeable TSL at the surface of arrested lesions greatly inhibits the rate of water evaporation and arrested lesions undergo smaller temperature changes during drying. Thermal imaging during dehydration with forced air was first used to detect lesions on extracted teeth [63,64]. Ando et al. [65] reported that pulse thermography is sensitive to the mineralization state of the tooth and can potentially be used for lesion activity assessment. The drop in temperature or thermal emission can be integrated over time during dehydration of the lesion to give a measure of the energy lost (ΔQ). Arrested lesions are expected to have lower values of ΔQ than active lesions. Thermography data can be used to obtain quantitative information on the extent of demineralization, such as lesion area, mineral loss and lesion depth, and ΔQ can be correlated with ΔZ [63]. Zakian et al. [64] showed that ΔQ maps compare well with lesion histology for occlusal lesions. Thermal imaging can also show differences in ΔQ between sound and active lesions [42,62]. Later studies suggested that thermal imaging is more sensitive on root surfaces than coronal surfaces [62]. Dentin has a higher water content than enamel, and the organic scaffold and dentinal tubules are mostly occupied by water when hydrated [66], and water retention in dentin increases with demineralization [67]. Lee et al. [62] showed that the ΔQ values for 18 active root lesions were significantly higher than for nine arrested lesions, 18.6 (18.7) vs. 0.7 (0.7), respectively. In the only clinical study employing thermal imaging, a micro-thermal camera operating at 8–13 µm wavelengths was used to monitor the thermal emission from suspected active root caries lesions during 30 s of air drying [61]. Lesions were also evaluated using CP-OCT before and after drying to resolve the lesion structure, detect the presence of a TSL and detect any shrinkage that may have occurred. Figure 4 shows an active lesion that has resulted in the collapse of the enamel near the CEJ, exposing a large area of the underlying dentin [61]. There are two thermal images shown in Figure 4B; the image after 30 s of air drying shows a localized dark area over the right portion of the lesion where the dentin is exposed. The lesion penetrates well under the enamel above and to the left of the exposed dentin. This can be seen in the two CP-OCT images of Figure 4C, taken at the two positions indicated by the arrows in Figure 4A. In the first image, the lesion has penetrated through the intact enamel near the gingiva and is also visible under the sound enamel (red dotted arrow) along the DEJ, shown by the light blue dotted arrow. In the deeper part of the lesion, where the enamel has collapsed, exposing the underlying dentin, it appears active in the thermal image and there is no surface zone visible in the OCT scan. There is a surface zone visible on the lesion in the first OCT scan in the position indicated by the dotted pink arrow, where the lesion has penetrated completely through the intact enamel. The areas of the lesion in the enamel with an intact surface zone and the areas of the lesion under the enamel are not as active in the thermal images since fluids are inhibited from diffusing to the surface. Thermal and CP-OCT images were analyzed from twenty-five test subjects with suspected active root caries lesions before and after drying the lesion with air [61]. The mean ΔQ values were significantly different between sound tooth surfaces, suspected arrested lesion areas and suspected active lesions, demonstrating that thermal imaging is a promising approach for the clinical assessment of lesion activity on root surfaces. The thermal emission profiles from the lesion areas were distinctly different from the sound regions of the tooth, which suggests that it may be possible to extract information regarding dehydration kinetics to provide even better discrimination between sound, arrested and active lesion areas [62].

Pulsed photothermal [68] and photoacoustic [69,70,71] imaging methods have also been investigated for the detection of dental caries. These methods use pulsed lasers to generate heat inside the tooth and then map out the resulting transient temperature or acoustic wave profiles to detect changes in mineralization. These methods may have potential for monitoring lesion activity; however, these methods have not been extensively investigated. Silvertown et al. [72] used the canary system to study the remineralization of simulated caries lesions and rated the degree of remineralization using the canary number system used to rate lesion severity.

## 7. SWIR Reflectance Measurements

Since enamel is highly transparent at longer wavelengths beyond the visible range, those wavelengths are ideal for imaging caries lesions [6]. The near-infrared (NIR) range is defined as 700–1000 nm and the short-wavelength infrared (SWIR) range as 1000–2500 nm. Early studies referred to the entire range from 700 to 2500 nm as the NIR, and only in more recent studies has SWIR been used to specify the longer wavelength range.

The highest contrast of demineralization on occlusal and root surfaces occurs at longer SWIR wavelengths beyond 1400 nm and stains do not interfere beyond 1200 nm [6]. The magnitude of water absorption varies markedly at SWIR wavelengths from 1000 to 2500 nm due to two strong water absorption bands near 1450 and 1950 nm.

Multiple imaging geometries are feasible at SWIR wavelengths to image lesions on all tooth surfaces, including proximal lesions due to enamel transparency; however, SWIR reflectance imaging is most useful for lesion activity assessment [6]. Commercial NIR clinical imaging devices are now available using NIR transillumination and NIR reflectance imaging geometries [73,74]. However, imaging systems operating at longer SWIR wavelengths are potentially capable of better performance due to the higher transparency of sound dental hard tissues and the transparency of stains at wavelengths beyond 1200 nm [6]. Longer wavelengths with increased water absorption and reduced light scattering from sound tissues offer significantly higher lesion contrast for early detection and more effective monitoring of demineralization and changes in lesion structure with remineralization. SWIR reflectance imaging at wavelengths with increased water absorption, namely near 1450 and 1950 nm, appears to be most sensitive to changes in water content, which is advantageous for lesion activity assessment [6].

**Figure 5:**
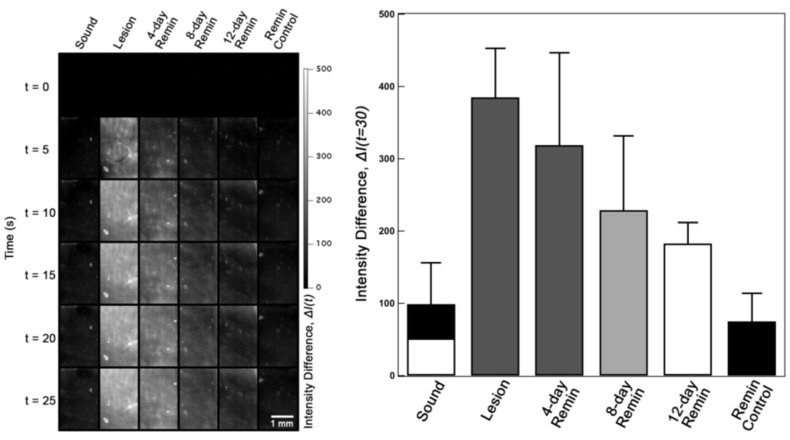
Intensity changes (ΔI) in reflectance at 1450 nm of bovine enamel surfaces during forced air drying after demineralization and remineralization. (**Left**) Images of the 6 treatment windows after different drying times. Note the largest degree of change occurs in the first 5 s between t = 0 and t = 5. (**Right**) Marked differences in (ΔI) were observed after remineralization. Bars of the same pattern are statistically similar (*p* > 0.05), *n* = 30. From reference [42].

The first dehydration studies using SWIR reflectance imaging were similar to those carried out using QLF, where the overall changes in the fluorescence loss (ΔF) were measured before and after dehydration [16]. These time sequences of SWIR images acquired at 1450 nm of a bovine enamel block with simulated lesions in six treatment windows, each of which had been exposed to varying degrees of remineralization, are shown in Figure 5 [42]. OCT scans indicated that a distinct TSL was formed on the lesion areas in each of the windows exposed to remineralization, reducing the porosity and permeability, arresting the lesion. Marked changes occurred in the reflected intensity difference, ΔI, before and after air drying (dehydration) and SWIR reflectance measurements were able to detect significant differences in ΔI between different periods of remineralization [42]. Most of the intensity change occurred in the initial 5 s, i.e., between t = 0 and t = 5 in Figure 5. Similar measurements on natural lesions showed a significant and marked decrease in the rate of dehydration if the lesion had a TSL visible with CP-OCT [42,62]. The largest changes in the reflected intensity occurred after drying near the 1450 and 1950 nm SWIR wavelengths that coincide with water absorption bands [43]. These studies were successful in showing large and significant differences in ΔI between simulated active and arrested lesions and shallow natural lesions on extracted teeth; however, later studies showed that this approach did not work well on deeper lesions on occlusal and root surfaces. Overall changes in reflectivity (ΔI) have been found to be sensitive to the depth and severity of lesions that cannot be established clinically before measurement [43].

Later studies have shown that more sophisticated methods that focus on the kinetics of dehydration rather than the overall change in reflectivity have proven to be more robust for assessing lesion activity. Dehydration kinetics appear to be primarily dependent on the influence of the highly mineralized surface zone that is present on arrested lesions, rather than the lesion depth and the severity of the lesion. Multiple parameters have been proposed to describe the kinetics or shape of the reflectivity curves, and the rise in intensity can be described by various equations, many of which were developed to describe enzyme kinetics [42]. Typically, the intensity rise takes a sigmoidal shape for active lesions. Dehydration curves of reflected light intensity vs. time are plotted in Figure 5 for various lesion types derived from two recent studies [75]. Defects due to hypomineralization or fluorosis typically have a similar structure to arrested lesions with a large TSL and have similar dehydration kinetics [75]. SWIR imaging at wavelengths greater than 1400 nm provides high-contrast images of fluorosis, which is valuable for monitoring the severity for epidemiological studies [75]. For active lesions on enamel surfaces, there is typically an initial delay before a rise in reflectivity occurs in SWIR reflectance images during drying because the water in the pores near the lesion surface initially absorbs all the incident light. After the water is lost, there is a rapid/steep rise in reflectivity that quickly reaches a plateau in intensity since the water in the body of the lesion can more rapidly escape through open pores. For arrested lesions, an initial delay before the rise in reflectivity is not anticipated because the highly mineralized surface layer lacks porosity and water. Dehydration occurs slowly because the highly mineralized surface layer inhibits the diffusion of water from the pores in the lesion body and the reflectivity rises slowly during drying.

**Figure 6:**
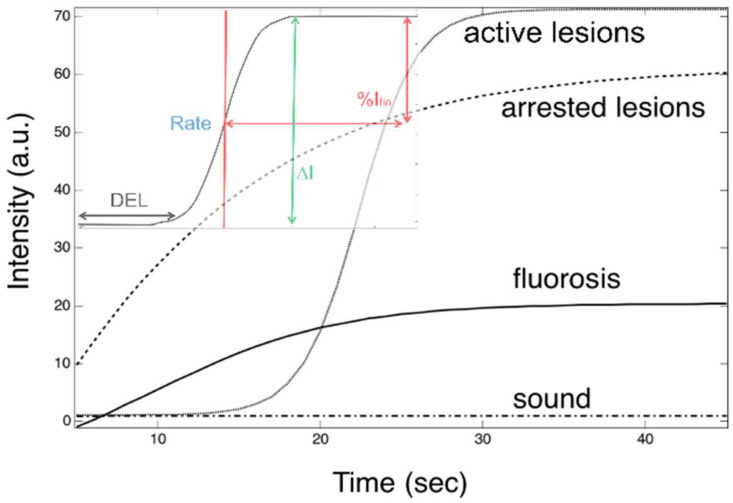
Mean reflected intensity vs. time curves plotted for sound, active and arrested lesions and areas of fluorosis during dehydration. The inset in the upper left corner shows how DEL, Rate, ΔI, and %I_fin_ are calculated from the curves.

In addition to ΔI, three more parameters have been used to describe the kinetics of lesion dehydration that vary markedly between active and arrested lesions [42,43]. These parameters are defined in Figure 6; DEL represents the initial delay between the time air is applied and the rise in intensity, the Rate represents the rate of increase in intensity once the rise begins and %I_fin_ represents the fraction of the intensity rise that occurs after the most rapid change in intensity occurs. These are empirical parameters that represent the shape of the rise in intensity of the dehydration curves. In the most recent studies, the rate of the rise in intensity of the s-shaped dehydration curves was calculated using the Hill equation developed to describe sigmoidal-shaped growth curves, as shown in Equation 1 [42,43]. I*_t_*_0_ is the intensity at time zero, the beginning of the curve, I*_end_* is the intensity at the end of the curve, and t_0.5_ is the time at which the intensity is at (I*_t_*_0_ + I*_end_*)/2. The parameter to be extracted is the Rate determined by the best fit to the Hill equation, as given by Equation (1).

(1) $$\;I\left(t\right)=I_{t0}+\frac{\left(I_{end}-I_{t0}\right)}{1+{\left(\frac{t_{0.5}}{t}\right)}^{Rate}}$$

The second kinetic parameter (%I*_fin_*) is simply the fraction of the intensity change that takes place in the tail end of the curve after the initial rapid rise in reflectivity. %I_fin_ is calculated using Equation (2) from the curve fitted using the Hill equation.

(2) $${\;\;\%I}_{fin}=\frac{\left(I_{tmc+a}-I_{t0}\right)}{\left(I_{tend}-I_{tmc+a}\right)}$$

where I_t0_ and I_tend_ are the intensities at the beginning and end of the curves and I_tmc+a_ is the intensity at the time of maximum change that is determined by taking the derivative of the dehydration curve (dI/dt) with an additional number of seconds (a) added dependent on the overall dehydration time.

Those four parameters were compared to the lesion depth and the surface zone thickness measured using microCT for 20 lesions on enamel smooth surfaces [43]. Lesion activity was based on the presence of a highly mineralized surface zone in microCT and a TSL in OCT images. A multivariate comparison of DEL, Rate and %I_fin_ was able to clearly distinguish between ten active and ten arrested smooth-surface coronal lesions, even though the overall mean for ΔI was similar [43]. The use of multiple complementary parameters to define lesion activity is a more robust approach than use of a single parameter.

Dehydration measurements from 1300 to 1950 nm show remarkable variation with wavelength [43]. Examination of dehydration curves at multiple wavelengths suggests that at 1450 and 1950 nm, water absorption predominates over light scattering [43]. Better discrimination between active and arrested lesions is feasible if multispectral dehydration curves with large differences in water absorption are simultaneously acquired, and two small studies on coronal and root surfaces support this hypothesis. [76]. In the first study, a reflectance imaging system employing a single photodetector was used to acquire time vs. intensity curves simultaneously at 1300 and 1950 nm for 30 lesions on tooth proximal and occlusal surfaces [76]. The absence or presence of a highly mineralized surface zone measured with microCT was used to indicate lesion activity. The 1950 nm curve was subtracted from the 1300 nm curve to generate a 1300/1950 difference curve and the exponential decay constant for the exponential fit of the difference curve (Diff DC) was calculated [76]. The ratio of means between active and arrested lesions on coronal surfaces was 38 for Diff DC compared to 3.4 calculated for the Rate that was derived solely from the 1950 nm curves, which is a 4-fold increase in discrimination. The larger mean was divided by the smaller mean for each parameter to yield the ratios. In a second pilot study, carried out at 1050 and 1450 nm using an InGaAs imaging array, the activity of 20 lesions, 10 active and 10 arrested, on root surfaces was assessed using this new differential absorption approach [77]. In that study, DIFF DC showed large and significant differences between DIFF DC between active and arrested lesions on root surfaces and the ratio of means was 7.9. Thermal imaging measurements were also carried out on the same root surfaces and ΔQ was calculated for comparison. The ratio of means was 8.4 for ΔQ, which was only slightly higher. This was the first study to show that SWIR reflectance imaging can also be used to assess the activity of lesions on root surfaces. Previous studies have shown that only thermal imaging was effective on root surfaces. These two studies demonstrate that SWIR reflectance imaging at two wavelengths with differential water absorption not only offers improved performance compared to previous methods but also performs well on root surfaces where previous methods of analysis were not effective.

SWIR wavelengths beyond 1400 nm yield a markedly higher contrast of lesions on tooth surfaces; however, they present additional clinical challenges due to increased water absorption. At wavelengths greater than 1400 nm, wet tooth surfaces can appear very dark and it is difficult to bring tooth surfaces into focus before they are dry. By using a second wavelength, such as 1050 nm, tooth surfaces are more visible when wet, which is an additional advantage of using two wavelengths.

There has only been one clinical study using SWIR reflectance imaging to assess lesion activity. Lesions on primary teeth were monitored for a period of six months during treatment with flouride varnish using CP-OCT and SWIR reflectance imaging [44]. Primary teeth with early lesions were selected to ensure that a high percentage of the lesions would be initially active. Images were acquired using CP-OCT every three months for 6 months [44]. SWIR reflectance images at 1400–1750 nm were acquired during forced air drying of the lesions for 30 s at 0 and 6 months. Most of the 42 lesions appeared initially active and only six lesions appeared initially arrested based on the presence of a TSL in the initial CP-OCT images. At 6 months, 14 of the lesions appeared arrested, including the six initially arrested lesions, and the mean TSL thickness increased significantly. The mean lesion depth and the integrated reflectivity over the lesion depth (ΔR) increased significantly after 6 months for the 42 lesions. Analysis of the dehydration curves from the SWIR videos acquired at 0 and 6 months yielded values for DEL and ΔI. The measured delays (DEL) were significantly higher (*p* < 0.05) for those lesions without a measurable TSL vs. those with a TSL. Due to the low number of lesions with TSLs, images from months 0 and 6 were pooled together to yield eight arrested videos and 40 active videos. For the mean ± s.d. of DEL, there was a large and significant difference (*p* < 0.05), 16 ± 8.9 s vs. 4.3 ± 5.1 s for active vs. arrested lesions. For active lesions, (ΔI) was higher, 18 ± 11 vs. 13 ± 11 for active vs. arrested lesions, but the difference was not significant [44]. These results are encouraging for the first clinical SWIR imaging study [44]. However, further in vitro studies have demonstrated the advantage of measuring multiple kinetic parameters derived from the dehydration curves for more definitive assessments of lesion activity and the assessment of lesion activity on all tooth surfaces [43].

## 8. Raman Imaging

Raman spectroscopy and imaging have been extensively investigated for caries detection and for the analysis of the mineral content of enamel and dentin [17]. Raman spectroscopy can measure spectral shifts in laser light scattered from tooth surfaces due to the molecular vibrations in the structure of enamel and dentin. Enamel spectra contain bands due to phosphate and carbonate while dentin also contains amide bands due to collagen content [78]. The spectra are dominated by the very strong phosphate band at 959 cm^−1^. The state of mineralization or the degree of crystallinity can be related to the width or area of the bands, ratios of bands and the intensity of the B-type carbonate band that changes markedly with caries. Maps of the tooth surface can be acquired by scanning a laser over the tooth surface [79,80,81,82,83,84]. Raman spectroscopy and imaging can be applied in vivo and there are several approaches, including spontaneous Raman scattering and stimulated Raman scattering, which have been used on tooth surfaces [85]. Polarization-dependent measurements can be used to enhance the differentiation of sound and demineralized enamel by measuring the polarization anisotropy or depolarization ratio of the most intense phosphate peak [86,87]. Less crystalline areas of demineralized enamel show a lower degree of polarization anisotropy. Wang et al. [87] showed that high-resolution polarization-resolved hyperspectral stimulated Raman scattering imaging could show differences between sound enamel and the lesion body and the surface zone. Therefore, this approach may be able to show differences in lesion activity.

Micro-Raman spectroscopy has also been used to assess the severity of fluorosis in vitro, since enamel crystallinity decreases with the increasing severity of fluorosis in a similar fashion to the severity of demineralization, and changes to B-type carbonate and phosphate bands can be measured [88,89,90,91].

Raman spectroscopy is capable of showing differences in crystallinity between sound and carious dental hard tissues; therefore, it is promising as a tool to assess lesion activity, and it can be combined with other methods such as OCT [86]. However, there have not yet been any studies in vivo or in vitro using Raman spectroscopy to assess lesion activity.

## 9. Limitations and Future Directions

Several optical methods have been investigated for assessing lesion activity, ranging from OCT to thermal imaging. Ideally, assessment should require only a single rapid measurement using a low-cost imaging system without the need to apply external agents. The system should also be capable of caries detection and the assessment of lesion severity and activity on all accessible tooth surfaces. None of the approaches discussed meet all those criteria. Four of these methods, for which there has been at least one clinical validation study, are summarized in Table 1. OCT appears to be the most promising approach, meeting all the criteria except for low cost, and has the unique advantage of providing high-resolution images of the subsurface lesion structure with greater than 10-micron resolution, similar to TMR and microCT, which is the closest to a gold standard for lesion activity. The high cost and the lack of any clinical systems on the market are major disadvantages of OCT. The other three dehydration imaging approaches suffer from the need for longer imaging times and concern about motion during imaging. QLF and thermal imaging have the advantage of being potentially low cost; however, it is not yet clear whether such systems can function effectively on all tooth surfaces. QLF is unlikely to work for root caries and suffers interference from stains, and it is not clear whether thermal imaging can work on tooth occlusal surfaces, where most new caries lesions are discovered. SWIR reflection imaging at wavelengths with higher water absorption appears to be most promising approach, and it also has great high potential for caries screening. The kinetic approaches used to interpret the SWIR dehydration curves give better discrimination between active and arrested lesions. Such kinetic approaches may also be feasible for use with QLF and thermal imaging to improve discrimination.

A major concern regarding all these new technologies is the lack of multicenter clinical validation. As can be seen in Table 1, there have been only a handful of clinical validation studies that have specifically focused on the assessment of lesion activity, and most have been from the author’s laboratory. There have been multiple studies of each technology from other research groups that have focused on assessing the severity of caries lesions rather than specifically addressing lesion activity, and those studies support the potential utility of the methods presented. For example, there have been only three clinical studies specifically validating the use of OCT for the assessment of lesion activity; however, there have been many studies both in vivo and in vitro showing that the structure of caries lesions can be resolved with OCT, and those show high-resolution images of the lesion structure, including the TSL at the lesion surface. Motion artifacts are a concern with all the technologies presented, especially the dehydration studies that require extended acquisition times of 5–30 s. Imaging probes can be placed in contact with tooth surfaces to inhibit movement and image registration algorithms are readily available for motion correction. Moreover, since none of these methods use ionizing radiation, if excessive movement occurs, the imaging can simply be repeated. The presence of saliva and water vapor is not a problem for OCT since it does not interfere at 1300 nm. It is a benefit since it reduces surface reflections and prevents dehydration that increases internal light scattering. In the case of dehydration imaging, the forced air rapidly removes any surface water and removes the interference of variations in water vapor above the tooth surface. Stains and plaque can significantly interfere with QLF imaging, as mentioned in Section 4, but they do not interfere with the other methods, which is a major advantage.

Both OCT and SWIR reflection imaging require the use of higher-cost SWIR imaging arrays that have hindered commercial development. However, the higher costs for SWIR InGaAs imaging arrays have been due to limited production and restrictions on the use of technology with military applications. Prices are dropping rapidly, with increased competition and the introduction of many nonmilitary applications, including medical thermography, firefighting, enhanced vision, security/surveillance, and machine vision. High-resolution thermal imaging devices that were once prohibitively expensive for personal use have dropped in cost by orders of magnitude over the past three decades and are now relatively inexpensive. New SWIR imaging technologies such as quantum dots that use alternative semiconductor materials and InGaAs are under development, and they offer higher performance at lower cost than InGaAs for large imaging arrays and have improved sensitivity at SWIR wavelengths beyond 1700 nm [92].

Artificial intelligence (AI) and machine learning have demonstrated great potential for caries detection, risk assessment and management [93,94,95,96,97]. Studies have included the analysis of optical images in addition to radiographs [97,98,99]. AI methods can likely be applied to all of the various methods proposed for assessing lesion activity to enhance performance. For example, AI can likely be used to process dehydration kinetics data, since several parameters can be extracted from the curves and combinations of multiple parameters can be analyzed to improve performance.

The application of pH sensing agents and Ca fluorophores also appears promising, since they may potentially be used to give a quantitative measurement of the degree of activity, but it needs to be demonstrated whether these agents can be used effectively in vivo. Tooth surface geometry, lesion severity, and stains may profoundly influence measurements, as they do for other methods.

Accurate determination of the degree of lesion activity and severity are of increasing importance for accurate diagnosis and effective clinical management and decision-making. It is well known that existing conventional methods are lacking and that new methods are needed to assess lesion activity and avoid unnecessary removal of the tooth structure. The advantages of new optical imaging methods that can show the lesion structure and the optical and physical changes between active and arrested lesions have been presented; they also offer the potential for lesion activity assessment during a single examination.

## Figures and Tables

**Table 1:** Summary of photonic methods for assessing lesion activity including cost, imaging speed in seconds, the surfaces that can be imaged (occlusal (O), root (R), and smooth (S)), and whether or not the method is able to resolve the internal lesion structure or is limited to only the surface. The number of clinical studies that have been carried out using that method is also listed.

Method	Cost	Speed	Surfaces	Structure	Clinical
OCT	very high	1–3	O, R, S	depth-resolved	3
SWIR	high	5–30	O, R, S	surface	1
Thermal	medium	5–30	R	surface	1
QLF	low	5–30	S	surface	1

## Data Availability

No new data were created or analyzed in this study.
